# Characteristics of the phenotypes in prevalent and incident cases of heart failure in primary care: IBERICAN study

**DOI:** 10.1186/s12875-024-02506-1

**Published:** 2024-07-25

**Authors:** Sergio Cinza-Sanjurjo, Miguel Ángel Prieto-Díaz, Vicente Pallarés-Carratalá, Rafael M. Micó-Pérez, Sonsoles Velilla-Zancada, Alfonso Barquilla-García, Leovigildo Ginel-Mendoza, Antonio Segura-Fragoso, Vicente Martín-Sánchez, José Polo-García

**Affiliations:** 1 CS Milladoiro, Área Sanitaria Integrada Santiago de Compostela, Travesía do Porto, Ames, 15895 PC Spain; 2 Instituto de Investigación Sanitaria de Santiago de Compostela (IDIS), Choupana s/n, Santiago de Compostela, 15706 PC, A Coruña Spain; 3 Centro de Investigación Biomédica en Red-Enfermedades Cardiovasculares (CIBERCV), Av. Monforte de Lemos, 3-5. Pabellón 11. Planta 0, Madrid, 28029 Spain; 4 Vallobín-La Florida Health Centre, Oviedo, Spain; 5 Department of Medicine, Jaume I University, Castellon, Spain; 6 Fontanars dels Alforins Health Centre, Xàtiva–Ontinyent Department of Health, Valencia, Spain; 7 Joaquin Elizalde Health Centre, Logroño, La Rioja, Spain; 8 Trujillo Health Center, Cáceres, Spain; 9 Ciudad Jardín Health Center, Málaga, Spain; 10 Epidemiology Unit, Semergen Research Agency, Madrid, Spain; 11 Gene-Environment-Health Interaction Research Group (GIIGAS), University of León, Leon, Spain; 12 Institute of Biomedicine (IBIOMED), Epidemiology and Public Health, Networking Biomedical Research Centre (CIBERESP), Madrid, Spain; 13 Casar de Cáceres Health Centre, Cáceres, Spain

**Keywords:** Heart failure, Diagnosis, Incidence, Primary care prevalence

## Abstract

**Background:**

The management in primary care (PC) of the patients with Heart Failure (HF) is different from the management hospital, in a special way compared to cardiology departments.

**Objective:**

To define the characteristics in both phenotypes of HF in prevalent and incident cases of HF in patients recruited in a large PC sample.

**Methods:**

We proposed a and longitudinal analyses, in patients of the IBERICAN cohort, that recruited 8,066 patients in the Spanish primary care system, with 15,488 patients-years of follow-up. Of them, 252 patients (3.1%) had diagnoses of HF. HF was classified according to the 2014 guidelines in two groups: HF with a reduced eject fraction or HFrEF (LVEF < 50%) and HF with preserved eject fraction or HFpEF (LVEF ≥ 50%). Recommended treatment was defined as the patient receiving drug treatment with Renin-Angiotensin-System (RAS) blockers with beta-blockers and, optionally, spironolactone. The incidence of new cases of HF was calculated in the 7,814 patients without HF in the inclusion visit. Finally, we analysed which variables associated the onset new cases and get the hazard ratio (HR) with the confidence interval at 95% ([95%CI]). Clinical trials register: NCT02261441 (02/05/2017).

**Results:**

The HFpEF was the most frequent phenotype in prevalent cases (61.1%) and incident cases (73.9%). Patients with HFrEF had a higher prevalence of coronary heart disease (*p* = 0.008) and PAD (*p* = 0.028), and no statistically significant differences was observed in the therapeutic groups used between both groups. The incidence of HF was 12.8 cases/1000 inhabitants/year, 35.6% of them was diagnosed in PC. The renin-angiotensin system blockers were more used in PC (60%) and beta-blockers (100%) and spironolactone (60%) in hospital. The female sex showed a protective effect for incident cases (0.51 [0.28–0.92]); and AF (HR [95%CI]: 2.90 [1.51–5.54]), coronary heart disease (HR [95%CI]: 2.18 [1.19-4.00]) and hypertension (HR [95%CI]: 1.91 [1.00-3.64]) increased the risk of developing HF.

**Conclusions:**

HF phenotype more frequent and incident in PC was the HFpEF, but only one third of them are diagnosed in PC level. The female sex showed a protective effect and atrial fibrillation, ischaemic heart disease and hypertension increased the risk of develop HF.

**Supplementary Information:**

The online version contains supplementary material available at 10.1186/s12875-024-02506-1.

## Introduction

In primary care (PC), heart failure (HF) is present in 3–5% of patients. Frequently, these patients visit the PC, emergency rooms, and hospitals due to comorbidities and polypharmacy. Exacerbations and hospital admissions of HF patients are associated with a poorer prognosis and increased mortality. The PC role, which combines physician and nurse work, may play a very important role in identifying exacerbations and adjusting treatment accordingly. Considering that the Guidelines recommend a first visit within fifteen days after discharge, in this sense, maybe a rapid response from a cardiologist or internal medicine would be advisable when a PC physician refers a patient [1]. In this way, HF is becoming into a paradigm for managing chronic diseases multidisciplinary.

Framingham defined and established the two main phenotypes still in use today: patients with reduced left ventricular ejection fraction (HFrEF) and patients with preserved left ventricular ejection fraction (HFpEF) [2]. There has been an increase in the prevalence of HF in recent decades, which correlates with a longer life expectancy, but also with greater prevalence of CVRFs, such as obesity and hypertension [3]. The greatest increase has been seen in patients with HFpEF [4], who have a lower readmission rate than HFrEF patients [5, 6] but similar mortality rates [7]. In patients with HF, the main role of PC is to control CVRF to avoid readmissions and to reduce mortality. This does not change the fact that it is unknown where the patients are diagnosed or what level of assistance is used to decide and prescribe treatment for them.

For a better understanding of the characteristics and management of patients with HF in PC, some of the most recent studies are CARDIOPRES [8] and EPISERVE [9], both of which have been in use for more than 20 years. These studies compared patients with HF in PC with patients in hospitals and provided information on their follow-up. The two studies describe the characteristics of patients in that moment, but with a different diagnosis and different management from hospital and PC departments in an older population and with more comorbidities, we don’t have new information to assess the situation [10]. HF represents the paradigm of the clinical situation that should be followed in PC: it is a chronic condition, increasingly prevalent, with frequent exacerbations in which a protocolized follow-up between the PC doctor and nurse [11] and a correct communication with the reference service that gives an adequate response in a short period of time [12], will enable the shared follow-up recommended by the guidelines [10].

The IBERICAN study, which was developed in Spanish PC, analyzes cardiovascular risk, recording CVRFs and cardiovascular diseases (CVD) among patients who are attended by their PC physicians [13]. HF is one of these CVD, and its phenotype is recorded in the study. With this information, we can describe the characteristics of the patients at this assistance level and analyze where they are diagnosed and treated.

The objective of our study is, based on the information available in the IBERICAN study, to define the characteristics in both phenotypes of HF in prevalent and incident cases of HF attended in PC.

## Materials and methods

### Study design and sample

We have analysed a transversal and longitudinal analysis in the patients included in the IBERICAN study is proposed attending to the HF diagnosis at the inclusion visit [13]. IBERICAN study is a cohort study approved by the Ethics Committee for Research with Medicines of the Hospital Clínico San Carlos of Madrid on February 21, 2013 (C.P. IBERICAN-C.I. 13/047-E) and registered at https://clinicaltrials.gov with the number NCT02261441 (02/05/2017).

The main objective of IBERICAN study is to analyse the prevalence and incidence of CVRF and CVD in the patients attended in the Spanish PC.

The sample was recruited consecutively, between 2014 and 2018, in PC offices, selecting patients between 18 and 85 years of age who met the following inclusion criteria: (1) user of the National Health System, (2) residing in Spain during the last 5 years, (3) registered with the physician researcher, and (4) signing the informed consent form and, who did not present any of the exclusion criteria: (1) change of habitual residence to another town or country within the next 6 months, (2) terminal illness or reduced life expectancy within the next 5 years, (3) evident difficulty to be followed up in PC, and (4) refusal by the individual to be part of the cohort in the first place or to continue to be part of it in the follow-up. The estimation of the sample size has been detailed in previous publications [13].

For this work, all patients were included in the analysis (*n* = 8,066), of whom we currently have follow-up data of 5,682, with a median of 26 months, which provides 15,488 years of follow-up (mean of 2.7 years/subject). 252 patients had previous HF at the inclusion visit, and we analysed the prevalence and the characteristics of both phenotypes in this group. In the other 7,814 patients without HF in the inclusion visit we estimated the incidence of new cases of HF.

The patients were considered as diagnosis of HF when they had clinical signs and symptoms of HF and an echocardiogram study to classify in one of both phenotypes (HFrEF (left ventricular ejection fraction -LVEF-<50%) and HFpEF (LVEF ≥ 50%)) defined in 2014 by the European Guidelines [14]. The researcher only recorded in the electronic database the type of HF, according to the ejection fraction described by the cardiologist in the clinical record of the patient.

### Variables

During the inclusion visit, socio-demographic data of each patient were recorded (sex, age, habitat, level of education, family economic status, current employment situation), toxic habits (tobacco and alcohol consumption), family history of early cardiovascular disease (CVD) and personal history (HTN, diabetes mellitus -DM-, hypercholesterolemia, coronary heart disease -IHD-, atrial fibrillation -AF-, HF, peripheral artery disease -PAD-, cerebrovascular disease), clinical parameters (weight, height, body mass index -BMI-, waist circumference, systolic pressure, diastolic pressure, pulse pressure -PP- and heart rate), collection of information on the presence or not of each CVRF, as well as its treatment. As complementary tests, lab tests (blood count, blood chemistry and urinalysis) and an electrocardiogram were recorded, which were considered valid if performed within the last 6 months before the inclusion of the patient.

HF was classified in two phenotypes according to the 2014 guidelines, when the IBERICAN study was launched: HFrEF (LVEF < 50%) and HFpEF (LVEF ≥ 50%) [14]. Following the same guidelines, *recommended treatment* for HFrEF was defined as the patient receiving drug treatment with Renin-Angiotensin-System (RAS) blockers with beta-blockers and, spironolactone. We cannot talk about optimized treatment because we do not know the doses of the drugs used [14]. The prevalent case of HF was defined when the patient had a previous episode with compatible signs and symptoms of HF and an echocardiogram to confirm one of both phenotypes (LVEF < 50% or LVEF ≥ 50%). The incident case was defined when a patient without previous HF diagnoses, develop a clinical episode with compatible symptoms and echocardiogram diagnoses of HF with anyone of both phenotypes. The echocardiogram was possible in hospitalized patients or in an ambulatory study in a patient referred by compatible symptoms. Cases of AF, IHD, or stroke leading to hospitalization or death were recorded at follow-up. In addition, a composite endpoint of admission or death due to CVD was defined, which included the presence of any of the CV diseases: HF, AF, IHD or stroke.

The outcomes considered in the follow-up were the main diagnosis in hospital admissions and mortality. The source of death cause used were the clinical report when the patient died in the hospital. For outpatient, the PC diagnosis or National Statistical Institute records were used.

### Statistical analysis

Quantitative variables with a normal distribution are presented as mean (standard deviation), while those without are presented as median (interquartile range [IQR]). The prevalence of HF was calculated using the data of the inclusion visit. Chi-square and T-student test were employed to compare percentages and means across both groups, respectively, for clinical outcomes.

The incidence was calculated using the follow-up visits in patients without previous diagnosis of HF. Subsequently, an analysis of these incident cases to establish in which setting the diagnosis and the start of treatment in these new cases were made. Finally, to analyse which variables associated the onset of these HF cases, a multivariate Cox analysis was performed in which age, sex, educational level, family economic level, alcohol consumption, smoking, physical activity, obesity, abdominal obesity, HTN, hypercholesterolemia, DM, IHD, AF and kidney disease were included as predictor variables. p-values below 0.05 were considered statistically significant results.

Statistical analyses were performed using SPSS version 22.0 (SPSS Inc., United States).

## Results

### Prevalent HF cases and their phenotypes

In the inclusion visit 252 patients (3.1%) had diagnoses of HF. Of them, 61.1% (*n* = 154) were diagnosed with HF with HFpEF and 38.9% (*n* = 98) with HFrEF.

The 52.4% of the patients with HF were women and 73.8% were over 65 years of age without differences between HFrEF and HFpEF (*p* = 0.161 and *p* = 0.105, respectively). The most frequent CVRF were HTN (79.4%) followed by hypercholesterolemia (70.2%) and DM (57.5%), with higher prevalence in HFrEF except HTN, but without reach statistical significance in anyone of them, Table 1. The most prevalent cardiovascular disease was AF (46.0%) followed by IHD (24.2%), with higher prevalence of IHD (*p* = 0.008) and PAD (*p* = 0.028) in patients with HFrEF, Table 1. The kidney disease did not show any differences between both groups, Table 1.

**Table 1:** Clinical and epidemiological characteristics in patients with heart failure, according to ejection fraction

	HFrEF		HFpEF		*p*
N	98		154		
Age					
18–44	7.4%	(3.01–14.44)	3.9%	(1.44–8.23)	0.105
45–64	25.3%	(16.90-34.87)	16.9%	(11.33–23.60)	
65 and older	67.4%	(56.98–75.96)	79.2%	(71.95–84.86)	
Women	47.4%	(37.02–57.33)	56.5%	(48.27–64.07)	0.161
**Cardiovascular Risk Factors**					
HTN	78.9%	(69.37–85.91)	81.2%	(74.08–86.54)	0.668
Hypercholesterolemia	71.6%	(61.40-79.66)	70.8%	(62.91–77.38)	0.892
DM	43.2%	(33.03–53.21)	40.9%	(33.06–48.81)	0.727
Obesity	51.6%	(41.09–61.38)	48.1%	(39.94–55.90)	0.589
Sedentary lifestyle	90.5%	(82.77–94.89)	89.0%	(82.91–92.97)	0.695
Smoking	11.6%	(5.92–19.57)	8.4%	(4.57–13.91)	0.415
**Cardiovascular disease**					
CHD	33.7%	(24.30-43.68)	18.8%	(12.98–25.75)	0.008
AF	46.3%	(36.02–56.31)	46.8%	(38.67–54.62)	0.946
Stroke	13.7%	(7.49–22.04)	12.3%	(7.59–18.47)	0.758
PAD	22.1%	(14.23–31.47)	11.7%	(7.08–17.73)	0.028
**Kidney disease**					
eGFR < 60 ml/min	29.0%	(20.08–38.97)	35.5%	(27.94–43.42)	0.294
ALB	18.8%	(10.08–30.02)	20.4%	(13.08–29.19)	0.772
Proteinuria	6.3%	(1.73–15.01)	3.9%	(1.07–9.55)	

HFrEF: heart failure with reduced ejection fraction; HFpEF: heart failure with preserved left ventricular ejection fraction; HTN: hypertension; DM: diabetes mellitus; CHD: coronary heart disease; AF: atrial fibrillation; PAD: peripheral arterial disease; eGFR: estimated glomerular filtration rate; ALB: albuminuria

The therapeutic groups used in both groups did not show differences between both phenotypes, Table 2.

**Table 2:** Drug treatments used in patients with heart failure, according to the ejection fraction

	HFrEF		HFpEF		*P*
N	98		154		
ACE inhibitors	41.3%	(30.07–52.67)	44.0%	(35.13–52.76)	0.712
ARBs	40.0%	(28.85–51.35)	42.4%	(33.61–51.18)	0.739
RAS Blockers	81.3%	(70.00-92.63)	86.4%	(77.24–95.19)	0.722
Beta-blocker	60.0%	(48.03–70.35)	49.6%	(40.53–58.25)	0.153
Diuretics					
Spironolactone	21.3%	(12.71–31.92)	12.8%	(7.49–19.80)	0.111
Loop diuretics	34.7%	(24.04–45.98)	34.4%	(26.13–43.10)	0.969
Thiazides	24.0%	(14.88–34.82)	19.2%	(12.70–27.00)	0.42
Statin	88.2%	(78.12–93.85)	79.8%	(71.05–86.25)	0.146
Metformin	61.0%	(44.50-74.37)	63.5%	(50.40-74.29)	0.796
SGLT2 inhibitors	4.9%	(0.59–16.16)	6.3%	(1.76–15.23)	0.753

HFrEF: heart failure with reduced ejection fraction; HFpEF: heart failure with preserved ejection fraction; ACE inhibitors: angiotensin-converting enzyme inhibitors; ARBs: angiotensin II receptor blockers; RAS: renin-angiotensin system; SGLT2 inhibitors: sodium-glucose cotransporter-2 inhibitors

### Outcome rates according to guideline-recommended treatment

Table 3 shows the incidence of events and the causes of death in the follow-up of HF prevalent cases. A higher rate of HF mortality (33.3% vs. 3.3%, *p* < 0.001), hospital admissions due to HF (16.7% vs. 11.9%, *p* = 0.727) and cardiovascular mortality (22.2% vs. 6.6%, *p* = 0.842) were observed in patients with no recommended treatment, but only HF-mortality reached statistical significance.

**Table 3:** Incidence of cardiovascular events in the follow-up of patients with heart failure with reduced eject fraction according to the optimization of drug treatment

	Recommended treatment	No recommended treatment	*P*
***N***	**6**	**92**	
**Hospital admission**			
Heart failure	11.9%	16.7%	0.727
Atrial Fibrillation	0.0%	6.6%	0.515
Coronary heart disease	0.0%	2.6%	0.686
Stroke	0.0%	2.0%	0.727
CVD	16.7%	21.9%	0.762
**Death**			
HF mortality	3.3%	33.3%	< 0.001
CV mortality	6.6%	33.3%	0.842

*HF: heart failure; CV: cardiovascular. CVD: cardiovascular disease*

### Incident HF cases

**Fig. 1:**
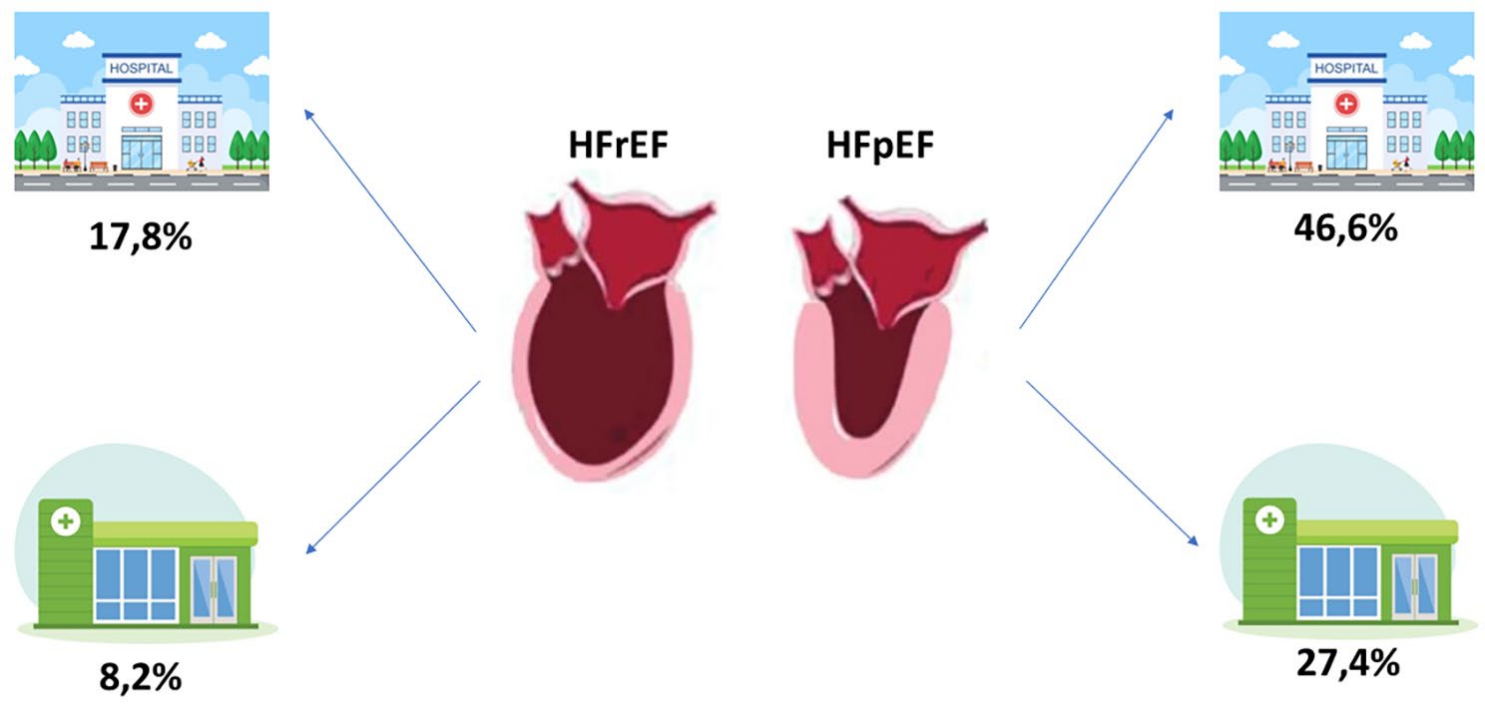
Incidence of each heart failure phenotype, and diagnostic setting. Food of figure: HFrEF: heart failure with reduced ejection fraction; HFpEF: heart failure with preserved ejection fraction; PC: diagnosis in primary care; H: diagnosis in hospital

Of the 7,814 patients without HF, during the available follow-up, 73 new cases of HF have been diagnosed (12.8 cases/1000 inhabitants/year), with a higher percentage of cases with HFpEF (*n* = 54; 73.9%), and 35.6% of cases were diagnosed in PC, Fig. 1.

The majority of patients were taken the recommended treatments before the diagnosis: ACE inhibitors or ARBs (93.2%), beta-blockers (97.3%) and spironolactone (93.2%). The beginning of new treatments with renin-angiotensin system blockers was the most frequent in PC (60%) and the start of beta-blockers (100%) and spironolactone (60%) were in hospital specialists.

**Fig. 2:**
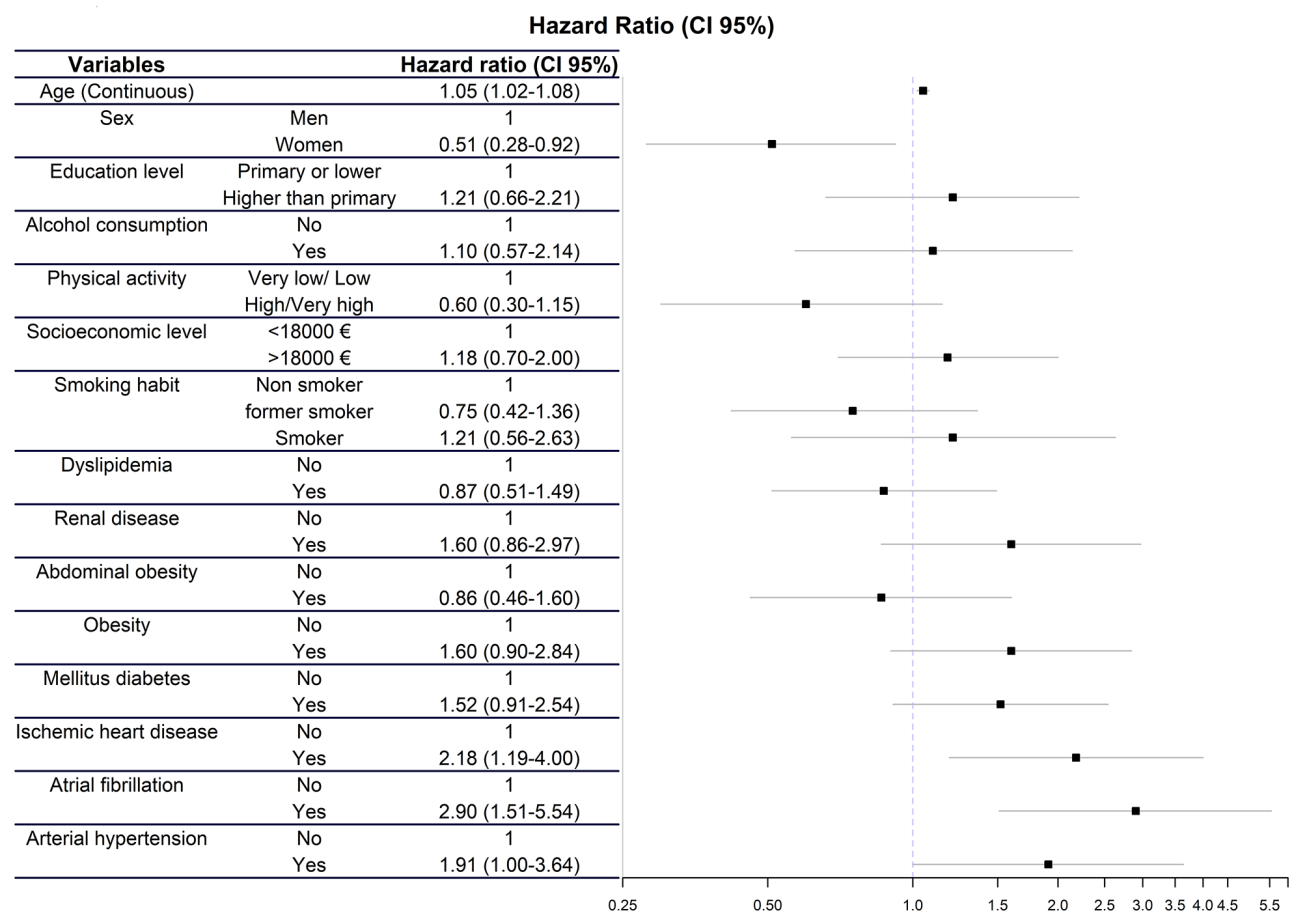
Multivariate Cox analysis to analyze the risk associated with each variable in the development of new cases of heart failure

The multivariate analysis showed that, for new cases of HF, female sex (HR [95%CI]: 0.51 [0.28–0.92]) behaved as a protective factor, and that AF (HR [95%CI]: 2.90 [1.51–5.54]), coronary heart disease (HR [95%CI]: 2.18 [1.19-4.00]) and HTN (HR [95%CI]: 1.91 [1.00-3.64]) was associated to new cases of HF, Fig. 2.

## Discussion

The results of our work, in a contemporary sampled recruited in PC, show that the most prevalent and incident HF phenotype in this assistance level is HFpEF. Among the new cases of HF, only a third of them are diagnosed in PC and the most frequent initial treatment in PC is RAS blockers. Most patients diagnosed with HFrEF do not receive the treatment recommended by the guidelines, but we have observed a trend to reduce HF mortality, HF admissions and CV mortality in patients who receive it, maybe because the number of patients with recommended treatment was reduced.

Our work, after an exhaustive literature review, is the newest that describes the clinical profile of prevalent and incident cases of HF in PC and complete these results with the description about the settings where is doing the diagnosis of the HF and the beginning of the treatment HF related. Our results are a first step to know what the actual situation in the diagnosis and treatment in the HF management is, but it is necessary to complete our results with a bigger sample to obtain more consistent results.

Our study shows similar prevalence of HF as other studies in Spain as EPISERVE (2%) [9] or EPICA (4.36%) [15] but higher than Italian (1.1%) [16] or Germany results [17], differences described previously [18]. Mayne the older people in Spain can explain these differences. Also, we have described that HFpEF is most frequent in PC. A difference in phenotypes frequency was described, with HFpEF more frequent in PC and other generalist specialists as internal medicine while the HFrEF is more frequent in cardiology departments [19], maybe the higher prevalence of IHD in the HFrEF explains this difference [9, 20–23].

Also, the incidence described in our study (12.8 cases/1000 inhabitants) is higher than other population-based studies in in Europe, between 1 and 9 cases/1000 inhabitants [24] and 3.44/1000 patients/year [25]. Our results also showed association of HTA and AF with the incident cases, both important causes of HFpEF [26, 27] and the coronary heart disease is associated with HFrEF [23]. This is a clear example of the importance of PC in CV prevention, with a correct control of CVRF and CVD as AF or chronic ischaemic heart disease, with a correct treatment to improve the prognosis.

Previous results published by our group described the low degree of adherence to the treatment scheme indicated by the guidelines [12] as other studies [28], in this work we extend the improved prognosis in patients who receive the 3 recommended therapeutic groups, even though this group only included 6 patients, and we have not enough statistical potence to confirm it. Also, our results showed a lower rate of use of loop diuretics (around 34%) and higher use of thiazides (around 22%) compared with other studies with data in the same years that showed (77.8% and 10.7%, respectively) [29]. Maybe these differences are related with higher use of thiazides in hypertension treatment and a lower use of loop diuretics in our sample to complain the Guidelines recommendations. The Guidelines in 2012, the current recommendations when our study began, indicated to reduce the diuretics according with an increase in mortality in patients with chronic consumption of these drugs [14]. Actually, the Guidelines define that we have to use the diuretics only in congestive situations [30].

However, we recognize some limitations in our study. We classified HF according to the indications of the 2014 guidelines, when the IBERICAN study began, so we do not have data on HFmrEF, which in our work is included in HFrEF. In our opinion, this is a minor limitation because it represents a small percentage of patients [31], and the classification used includes the current definition of HFrEF and HFmrEF. The actual guidelines recommend the same treatment in both phenotypes (HFrEF and HFmrEF) that we included in the HFrEF, and it defines similar risk factors, physiopathologically and prognosis in both situations [32]. There are other limitations published in previous manuscripts as the sample recruited in clinical practice, and it limits the external validation to populations. Other limitations that we want to highlight is that the physicians who participated in the study was not randomized, they participated voluntarily; and maybe they can manage better the patients because they have more knowledge about CV risk. Finally, we have to explain that our reduced sample size, specially in the incidence, does not let us to obtain definitely conclusions about the management of HF patients in PC, but our results are the most recent data about this disease, and it can show some idea about that.

For all the above, we can conclude that, HFpEF is the most frequent phenotype in PC. The diagnosis and the beginning of the treatment are doing in hospital assistance, maybe because the final test (echocardiogram) is doing in this assistance. The variables associated with the incidence of HF were AF, coronary heart disease and HTN. The female sex was associated with a protective effect.

### Electronic supplementary material

Below is the link to the electronic supplementary material.

Supplementary Material 1

## Data Availability

Data sharing is not applicable to this study.
