# Supplementary material for: Characteristics of the phenotypes in prevalent and incident cases of heart failure in primary care: IBERICAN study
Source: BMC Prim Care. 2024 Jul 25;25:271. doi: 10.1186/s12875-024-02506-1 (PMC11270967; doi:10.1186/s12875-024-02506-1)
Supplement: Supplementary file 1 — Supplementary Material 1 [file 12875_2024_2506_MOESM1_ESM.docx]

**SEMERGEN (Spanish Society of Primary Care Physicians) Foundation**

**Scientific Committee:** Alfonso Barquilla García, Ángel Díaz Rodríguez, Carlos Escobar Cervantes, Francisco Javier Alonso Moreno, Jesús Vergara Martín, Juan José Badimón, José Polo García, Luis Rodríguez Padial, Miguel Ángel Prieto Díaz, Rafael Vidal Pérez, Sergio Cinza Sanjurjo, Sonia Miravet Jiménez, Sonsoles Velilla Zancada, José Ramón Banegas, Vicente Martín Sánchez, Vicente Pallares Carratalá, Antonio Segura Fragoso y Rafael Manuel Micó Pérez.

**Andalusia:** Antonio López Téllez, Jesús Vergara Martin, María De Los Ángeles Ortega Osuna, Cristóbal Prieto Cid, Mª José Hidalgo Fajardo, José Lorente Serna, Ángel Domínguez Requena, Ricardo Alberola Cañizares, Manuel Ruiz Peña, Filomena Herrero Collado, Marcela Montes Vázquez, Rafael Ángel Carrascal Garrido, María Reyes Herrera Lozano, Beatriz Ortiz Oliva, Francisco José Anguita, Carmen Pérez Ibáñez, Carlos Alberto Cabrera Rodríguez, María José Cruz Rodríguez, Sandra Bonilla Ruiz, Rocío Reina González, Salome Abad Sánchez, Inmaculada Santana Martínez, Rafael Sánchez Jordán, Juan Mª Ramos Navas-Parejo, José Manuel Ramírez Torres, José María Beltrán Poveda, María Adoración De Cruz Benayas, Carmen Fernández Gil, Jon Iñaki Esturo Alcaine, Antonio Mora Quintero, Fernando Leiva Cepas, José Luis Carrasco Martín, Emilio García Criado, Mercedes Vázquez Blanco, Isabel Mora Ortiz, Leovigildo Ginel Mendoza, Juan Carlos Aguirre Rodríguez, Esperanza María Romero Rodríguez, José Acevedo Vázquez, Juan Gabriel García Ballesteros, María De La Paz Fernández Lara, Patricia Agüera Moreno, Eduardo Paños Maturana, Juan Manuel Ignacio Expósito, Noelia Carrillo Peñas, Carmen María Abad Faya, Ana Marina Almagro Duque, Rubén Torrescusa Camisón, Paloma Menéndez Polo, Marina Peña García, Cristina López Fernández, Ascensión Estepa Torres, Miguel Gutiérrez Jansen, Esperanza Loizaga González, Lisardo García Matarín, Enrique José Gamero De Luna, Javier Benítez Rivero, María José Gómez González, Carmen Gómez Montes, Juan Carlos Rodríguez Rodríguez, Juana María González Barranco, Josefa Ramírez Vizcaíno, María Ángeles Miranda Sánchez.

**Aragon:** Eva Trillo Calvo, Concepción Bayod Calvo, Susana Larripa De La Natividad, German Grasa Lambea, Emilio Jiménez Marín, Ana Cristina Navarro Gonzalvo, Antonio Pablo Martínez Barseló, Irene Peña León, Ángel González Pérez, Liliana Mahulea.

**Asturias:** María José Pérez Martínez, Ana Piera Carbonell, Margarita Alonso Fernández, María Montserrat Rueda Cuadrado, Rodrigo Abad Rodríguez, José Miguel Álvarez Cabo, Rubén Sánchez Rodríguez, Eva María Cano Cabo, Anny Romero Secin, Miguel Ángel Prieto Díaz, Juan Jesús García Fernández, Saúl Suárez García.

**Balearic Islands:** Fernando García Romanos, Antonia Moreno González, María Lara Amengual Sastre, Susana Martínez Palli, José Alfonso Ramón Bauza, Jose Ortiz Bolinches, Carmen Fernández Fernández, María Isabel Orlandis Vázquez, Ana Sanchis Mezquita, Fernando Unceta Aramburu, Juan Fernando Peiró Morant, Ana Moyá Amengual, Mateu Seguí-Díaz.

**Basque Country:** José Félix Zuazagoitia Nubla, Ana Echevarría Ituiño, Gregorio Mediavilla Tris, María Carmen Noriega Bosch, Esther González, María Luisa Ruiz Macho, Ruth Sendino Del Olmo, Asunción Olagorta De Prado, Ana López De Viñaspre Muguerza, Jesús Iturralde Iriso, Mª Rosario Virtus Iñurrieta, Lucas Ulloa Bahamonde.

**Canary Islands:** Isidro Godoy García, Fernando Rubio Sevillano, María Isabel González González, Marta Pérez Souto, Raquel De León Contreras, Sara Isabel Almeida González, Irene Almería Diez, Virginia María Mirabal Sánchez, Francisco Jose Escobar Lavado, Yoel Anta Pérez, Nayra Sánchez Hernández, Juan Luis Alonso Jerez, Ricardo Koch, Nayra Ramírez Mendoza, Héctor Suárez Hernández, Francisco Jesús Morales Escobar.

**Cantabria:** E. Lidia Gutiérrez Fernández, Fernando Andrés Mantecón, Ana Belén García Garrido, Asunción Vélez Escalante, Luisa Alonso Rentería, Jesús Sainz Jiménez, Guillermo Pombo Alles, Esperanza Rueda Alonso.

**Castilla La Mancha:** Juan Antonio Divisón Garrote, Pedro Martínez Sotodosos, Juan Antonio Vivancos Fuster, María García Palencia, José Ambrosio Torres Moraleda, Sara González Ballesteros, Ana Carmen Gil Adrados, Antonio González Cabrera, Miguel Ángel Babiano Fernández, Guillermo Rico García, Juan José Criado Alvarez, Pilar Torres Moreno, Francisco Javier Arribas Aguirregaviria, Alicia Sahuquillo Martínez, Lourdes María Santos Bejar, Miguel Laborda Peralta, Raúl Piedra Castro, Carlos Santos Altozano, Lucia González Tarrio Polo, Pedro Valiente Maresca, Reinilda Mota Santana, Noemi Elizabeth Terrero Ledesma, Noelia Garrido Espada, Francisco Javier Alonso Moreno, Gabriela Delia Rosa Zambrana Calvi, Cristina de Castro Mesa, Blanca Cordero García, Pilar Sorrius Sitges, Ana María de Santiago Nocito, César Lozano Suárez.

**Castile and Leon:** Juan Lorenzo Gutiérrez Montero, Juan Ignacio López Gil, María Dolores Fernández Ortega, Miren Elizari Roncal, María Ascensión López Serrano, Nuria Esther Adrián De La Fuente, Belén Angulo Fdez. De Larrea, Naiara Cubelos Fernández, Guiomar Luz Ferreiro Gómez, Diana Gómez Rodríguez, Sonia María Andrés Tuñón, María Ajenjo González, Serafín De Abajo Olea, Juan José León Regueras, César Manuel Gallego Nieto, Delio Vázquez Mallada, María De La O Gutiérrez García, Pablo Baz Rodríguez, José Ignacio Ferradal García, Blanca Delia De Román Martínez, Ana Arconada Pérez, Omar Mahmoud Atoui, Álvaro Morán Bayón, María Teresa Armenteros Del Olmo, Francisco Javier García-Norro Herreros, Enrique Méndez Rodríguez, Diana María Narganes Pineda, Ángel Díaz Rodríguez, Verónica Ortiz Ainaga, Milagros Sonlei Sánchez Guevara, Laura Villota Ferreiro, M Teresa Grande Grande, Francisco Vicente Martínez Gracia, Jesús Palomo del Arco.

**Catalonia:** María Dolores Moriano García, Beatriz Jiménez Muñoz, Gemma Rovira Marcelino, Diana Elizabeth Fernández Valverde, Roser Rodó Bernadó, María Teresa Ortiz Lupiañez, Najlaa Najih, José María Diéguez Parra, Mª Rosa Benedicto Acebo, Mari Luz Bravo Vicien, Alberto Ramón León Estella, Juan Antonio Muñoz Gómez, Alicia Mostazo Muntané, Isabel Ortega Abarca, Anna Gasol Fargas, Brenda Elizabeth Riesgo Escudero, Susana Elizabeth Riesgo, Edgar Zaballos Castellvi, Celia Cols Sagarra, Marta Herranz Fernández, Josep Alins Presas, Idaira Damas Pérez, Rosa M Alcolea García, Ines Monte Collado, Roberto Genique Martínez, María José Guasch Villanueva, Sònia Miravet Jiménez, Teresa Rama Martínez, Lucio Pinto Pena, Josefa María Panisello Royo, Inés Gil Gil, Carlos Gómez Ruiz, Rita Sahun Font, Anna Fuentes Lloveras.

**Community of Madrid:** Alberto Calderón Montero, María Del Mar Zamora Gómez, Elena Alarcón Cebrián, Mª José Piñero Acin, Celia Pecharroman Sacristán, M Soledad Mayayo Vicente, Mª Paz Pérez Unanua, Nuria Marañón Henrich, Saray Gómez Monreal, Sonia Redondo De Pedro, Blanca Sanz Pozo, Irene Moreno Martínez, Beatriz López Uriarte, Carmelina Sanz Velasco, Amaya Gárriz Aguirre, Montserrat Rivera Teijido, German Reviriego Jaén, José Ignacio Aza Pascual-Salcedo, Josefa Vázquez Gallego, Julia Caballer Rodilla, Aida Herrera, Ezequiel Arranz Martínez, Ana María Gómez Calvo, Paula Morán Oliva, Mª Milagros González Béjar, Julio Antonio Heras Hitos, Olga Garcia Vallejo, Manuel De Jesús Frías Vargas, María Jesús Castillejo Boguerin, Aurora García Lerín, Miguel Ángel María Tablado, Elena Concepción García García, Leticia De Miguel Acero, Carmen Zárate Oñate, Aránzazu Barranco Apoita, María Ester Montes Belloso, Ana Maria Huertas Velasco, Rafael Sáez Jiménez, Julia Natividad García Pascual, María Clemencia Zuluaga Zuluaga, Mª Cruz Díez Pérez, Antonio Ruiz García, Cristina Murillo Jelsbak, Virginia Lasso Oria, Amelia González Gamarra, Elena Rodilla Rodilla, Alberto Galgo Nafría, María Mestre de Juan, Mª Carmen García Albiñana, Mª del Pilar Moreno Cano, Paula Hernanz López, Paloma Casado Pérez.

**Estremadura:** Jacinto Espinosa García, José Ignacio Prieto Romo, Leandro Fernández Fernández, Javier Sierratapia, Nieves Moreno Regidor, Francisco Javier Zaballos Sánchez, Ana Moreno Moreno, Francisco Carramiñana Barrera, Juan José Torres Vázquez, María José Gamero Samino, Miguel Ángel De Santiago Rodríguez, Pablo Rafael Gómez Martínez, Antonio Carlos Elías Becerra, Javier Soto Olivera, Víctor Cambero, Julián Domínguez Ávila, Andrés Simón Fuentes, Jorge Manuel De Nicolás Jiménez, Dimas Igual Fraile, Guadalupe Nieto Barco, Ignacio Araujo Ramos, Mª Luz Serrano Berrocal, Francisco Buitrago Ramírez, Minerva Gallego Marcos, Félix Suarez González, Victoriano Chavero Carrasco, José Polo García, Francisco Guerra Peguero, Francisco Javier Sánchez Vega, Manuel Tejero Mas, Alba Palmerín Donoso, Miguel Turégano Yedro, María Beatriz Esteban Rojas, Fátima Cabezudo Moreno, Nawson Elver Quevedo Saldaña, María Del Mar García Fenés, Alfonso Barquilla García, Timotea Garrote Florencio, José María Fernández Toro, Vicente Caballero Pajares, María José Gómez Barquero.

**Galicia:** Alejandra Rey Rañal, Elena García Del Río, Enrique Nieto Pol, Julio Álvarez Fernández, Pilar Alonso Álvarez, Mª Luisa Jorge Gómez, Antonio Calvo Guerrero, Isabel Celemín Colomina, Lucia Barreiro Casal, Juana Fernández Moreno, Mª Angelines Carballal Martínez, Nabor Díaz Rodríguez, Carlos Moral Paredes, Dolores Recarey García, Francisco Javier Iglesias Mato, Antonio Fouz Ulloa, Amparo Fidalgo González, Noelia Dios Parada, Patricia Conde Sabarís, Ana Isabel Rodríguez Pérez, Ana Inés García Palacio, Víctor Julio Quesada Varela, Lidia Romero Iglesias, Ángel Lado Llerena, Carmen Lires Rodríguez, María Luisa Carretero Díaz, José Carreira Arias, José Luís Vázquez Camino, María Del Carmen Torreiro Penas, Sandra Yáñez Freire, Sergio Cinza Sanjurjo, Daniel Rey Aldana, Carlos Piñeiro Díaz, Portal González Lorenzo, José Rodríguez Campos, Rubén Blanco Rodríguez, Manuel Portela Romero, Lucía Vilela de Castro.

**Rioja:** Sonsoles María Velilla Zancada, Rafael Crespo Sabarís, Oscar Fernando Isaula Jiménez.

**Melilla:** Jesús Manuel González Puga, Jorge Antonio Benaín Ávila, Óscar Del Toro González.

**Murcia:** Vicente Llorca Bueno, Ana María Ballesteros Pérez, Domingo J. Rubira López, Mª Dolores Esteve Franco, Elena Sánchez Pablo, María Teresa Palacios López, Juan Castillo Meroño, José María Lobo Martínez, Isabel María Peral Martinez, J. Eduardo Carrasco Carrasco, Armando Santo González, Juan Gomáriz García, Beatriz Ríos Morata.

**Navarre:** Laura Sánchez Iñigo, Inés Sanz Pérez.

**Valencian Community:** Vicente Pascual Fuster, Mª Dolores Aicart Bort, Natividad Vázquez Gómez, Carlos Lluna Gasco, Teresa Amorós Barber, Pedro Antonio Medina Cano, Miguel Monteagudo Moncho, Mª Jesús Larré Muñoz, Raquel Navarro Hernández, Francisco José Martínez Egea, Antonio Tramontano, Marta Ferrer Royo, Belén Persiva Saura, Juan A. Contreras Torres, José Mª Tirado Moliner, Alejandro Salanova Penalba, Ariadna Cucó Alberola, Fernando María Navarro I Ros, Enrique Beltrán Llicer, Ana Seoane Novás, Inmaculada Martín Valls, Gracia Verdú Mahiques, Enrique Peña Forcada, Nieves Aguilar Gómez, Francisco Javier Sanz García, M Dolores Paradís Bueso, María Eugenia Alegre Romero, Antonio Francés Camus, María Amparo Anton Peinado, Rosa Latorre Santos, Mª Asunción Palomar Marín, María Carmen Botella García, Eva Sánchez Fresquet, Pedro Sala Paños, Tomás Sánchez Ruiz, Rosa Ana Valero Valero, María Seoane Vicente, Magdalena Martin Llinares, Antonio Masiá Alegre, José Luis Llisterri Caro, Irene Lluch Verdu, Vicente Pallarés Carratalá, Francisco Valls Roca, Rafael Manuel Micó Pérez, Carmen Barceló Dupuy, Elena Benages Vicente, María José Gimeno Tortajada, Mercedes Calleja del Ser, Martín Menéndez Rodríguez, Rosalía Victoria Carbonell Castelló.
